# Post Traumatic Superficial Temporal Artery Aneurysm: Highlighting the Importance in History and Examination

**Published:** 2014-12-30

**Authors:** Oliver Sawyer, Robert Staruch, Mohammed Ellabban

**Affiliations:** ^a^North Bristol Trust, Bristol; ^b^Royal Free Hospital, London, UK; ^c^University Hospital of Coventry and Warwickshire, Coventry, West Midlands, UK

**Keywords:** aneurysm, ligation, temporal artery, trauma, surgery

## DESCRIPTION

**Figure 1:**
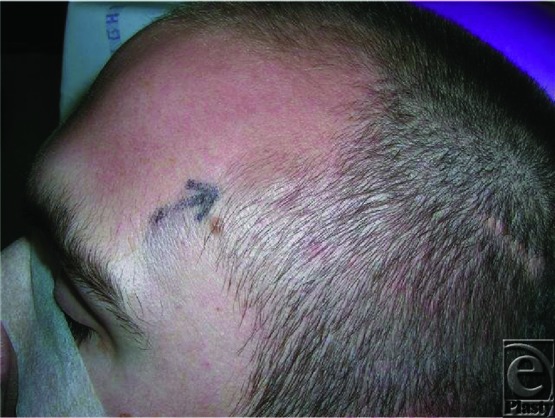
Preoperative appearance of aneurysm with position marked on the skin.

A 21-year-old carpenter received a blow to the left side of the head during a rugby match but sustained no wound or loss of consciousness. Three months later, he developed a mass on the left temple that expanded in size particularly after exercise and in warm environments (Fig 1).

## QUESTIONS

**Enter questions here; press return to add another question. Each Interesting case must have 4 questions**.

1. Describe the anatomical course of the superficial temporal artery (STA)?
2. What features in the history and examination would guide your diagnosis of aneurysm?
3. Describe the pathophysiology of a post traumatic aneurysm of the STA?
4. What is the management of an STA aneurysm?

## DISCUSSION

The STA has a fairly long and arduous anatomical course after its origin from the external carotid artery at the inferior pole of the parotid gland.^2^ Literature highlights the proximal branch of the STA, at the point at which it transverses the attachment of temporalis fascia to the superior temporal line, to be the area of vessel most susceptible to aneurysm. The reason for this pathological process is believed to be due to the shearing and crush forces associated with the projection of the bony ridge that acts as the origin of temporalis muscle in relation to the STA.^3^ Blunt trauma to the side of the head renders the artery vulnerable to significant crush injury against the bony projection.

A review of the literature identifies that diagnosis is largely based in most of the cases on effective patient history that reveals a recent episode of blunt, or possibly penetrating, trauma to the head. Furthermore, possibly more probing, questioning usually allows the patient to reveal or recall the incident between 2 to 6 weeks previously. Although the lesion itself is painless on examination, patients may incidentally report an associated headache since injury. This is followed by clinical examination, in uncomplicated cases, with particular attention to examine the mass in detail, noting size, consistency, adherence to underlying structures as traditionally taught. Experience and correlation with previous literature reports find that the common presentation is of a nonfluctuant, painless, solitary, expansile mass in the temporal and pterional region of the skull.^4^ In addition, examination may find superficial abrasions to suggest injury; however, in this case, there was no overlying skin injury. Specifically, clinicians should palpate the mass with simultaneous occlusion of the temporal artery proximally. This should result in obliteration of the pulse within the mass. This is helpful as it provides diagnostic confirmation of the etiology.

**Figure 2:**
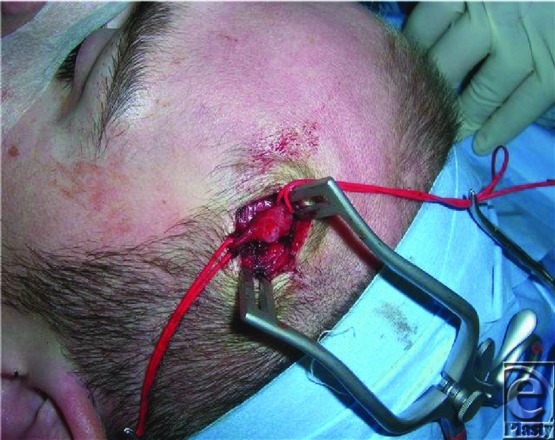
Intraoperative view of aneurysm prior to ligation.

**Figure 3:**
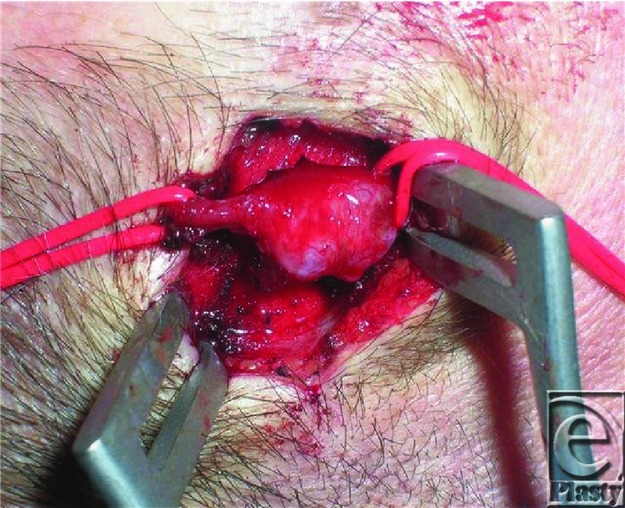
Close up view of STA aneurysm.

Typically the hematoma formed after injury to the vessel hardens and undergoes fibrosis forming a hard fibrotic capsule. This can often completely occlude the vessel lumen. Resultant luminal thrombosis and lysis allow recanalization.^5^ The fibrous scarring found surrounding the vessel can be substantial. The combination of scarring and recanalization of the thrombosis result in the increased dilated size (Figs 2 and 3).

Although much aneurysmal disease often requires fast and immediate treatment, patients suffering from an STA aneurysm can often be brought back for elective surgery and treatment. The risk of local tissue necrosis, damage to local organs (the vestibular center), and possible nerve injury mean that intervention is preferred to prevent serious long-term complications.^5^ Current surgical management of this condition consists of dissection of the scalp to reveal the defect followed by simple excision and ligation, this is considered the “gold standard” of treatment for STA aneurysms.^1^^,^^3^^,^^4^ Conservative management consists of compression of the hematoma itself, which experience has shown to be relatively ineffective, close observation as well as appropriate analgesia is more appropriate. Some studies have shown limited successes of endovascular repair of this vessel.

Superficial temporal artery aneurysm is a classic example where thorough history and careful examination can provide an accurate diagnosis without expensive or invasive imaging and investigation. It is a condition plastic surgeons should be aware of and capable of managing when similar cases present.

## References

[1] Cheng CA, Southwick EG, Lewis EC (1998). Aneurysms of the superficial temporal artery: literature review and case reports. Ann Plast Surg.

[2] Pipinos II, Dossa CD, Reddy DJ (1998). Superficial temporal artery aneurysms. J Vasc Surg.

[3] Harris KA, Walker PM, Hardacre GA (1983). Post-traumatic aneurysms of the superficial temporal artery. Can Fam Physician.

[4] Shenoy SN, Raja A (2003). Traumatic superficial temporal artery aneurysm. Neurol India.

[5] Benoit B, Wortzman G (1973). Traumatic cerebral aneurysms clinical features and natural history. J Neurol Neurosurg Psychiatr.

